# The Effects of Aging on the Components of Auditory – Verbal Short-Term Memory

**DOI:** 10.5334/pb.bm

**Published:** 2015-12-08

**Authors:** Clémence Verhaegen, Martine Poncelet

**Affiliations:** 1 Department of Psychology: Cognition and Behavior, University of Liège, Liège, Belgium

**Keywords:** Elderly participants, auditory-verbal short-term memory, item information, order information, hearing loss

## Abstract

This study aimed at exploring the effects of aging on the multiple components of the auditory-verbal short-term memory (STM). Participants of 45–54, 55–64, 65–74 and 75–84 years of age were presented STM tasks assessing short-term retention of order and item information, and of phonological and lexical-semantic information separately. Because older participants often present reduced hearing levels, we sought to control for an effect of hearing status on performance on STM tasks. Participants’ hearing thresholds were measured with a pure-tone audiometer. The results showed age-related effects on all STM components. However, after hearing status was controlled for in analyses of covariance, the age-related differences became non-significant for all STM processes. The fact that age-related hearing loss may in large part explain decreases in performance on STM tasks with aging is discussed.

## Introduction

The question of whether there is a decline in auditory-verbal short-term memory (STM) in older adults has received considerable attention (e.g., Belleville, Peretz, & Malenfant, 1996; Bopp & Verhaeghen, 2003; Fisk & Warr, 1996; Grégoire & Van der Linden, 1997; Hester, Kinsella, & Ong, 2004; Kaulser, 1994; Peters et al., 2007). In these studies, STM has generally been assessed using span tasks, following the phonological loop model of Baddeley and Hitch (1974). In these tasks, participants are presented with series of stimuli (digits, letters or words) and then asked to repeat these items in the order in which they were presented. A large number of studies and reviews have reported significant decline in STM spans with aging (e.g., Grégoire & Van der Linden, 1997; Hester et al., 2004; Kaulser, 1994). In their review of STM decline in aging, Bopp and Verhaeghen (2005) indicated that older adults’ retention of memoranda in short-term serial recall tasks is about 90 percent of that of younger adults. However, some studies have found no age-related decline on serial recall tasks with words or digits (e.g., Belleville et al., 1996; Fisk & Warr, 1996; Peters et al., 2007) and the question of whether STM declines in the elderly is still a matter of debate.

According to more recent conceptions of STM, participants have to store two types of information in these typical auditory-verbal STM tasks: item information, which consists of the phonological and lexical-semantic characteristics of the items presented, and serial order information, which concerns the order in which the items are presented (see Majerus, 2013, for a review). A number of studies have suggested that item and order information are dealt with by distinct mechanisms and stored separately. These components could be selectively impaired, and thus should be assessed separately. Item information is thought to be maintained via temporary activation of phonological and lexical-semantic language representations, while order information is said to be stored via a specialized processing module and represents what one might consider as being the remaining specific property of STM processing (Burgess & Hitch, 1999; Gupta, 2003; Henson, 1998; Majerus, 2009; Majerus, Heiligenstein, Gautherot, Poncelet, & Van der Linden, 2009; Majerus, Leclercq et al., 2009; Majerus, Poncelet, Elsen, & Van der Linden, 2006; Majerus, Poncelet, Greffe, & Van der Linden, 2006; Majerus, Poncelet, Van der Linden, & Weekes, 2008). This distinction is based on a number of empirical studies showing dissociations between STM capacities for the retention of item and order information. For instance, experimental studies on healthy adults using immediate serial recall tasks have shown that linguistic knowledge (lexical frequency or semantic similarity) substantially affects item recall, but has a much lesser effect on order recall (e.g., Nairne & Kelley, 2004; Saint-Aubin & Poirier, 1999). Moreover, neuropsychological studies have also demonstrated that item and order STM can be selectively impaired. For instance, Majerus, Norris, and Patterson (2007) showed that two patients with semantic dementia (i.e., a progressive loss of semantic representations), AT and WM, presented impaired item STM performance but preserved order STM capacity. Similarly, Attout, Van der Kaa, Georges, and Majerus (2012) showed a double dissociation between item and order STM deficits in two brain-damaged patients, MB and CG. On tests of STM capacity, MB showed poor item STM performance but preserved order STM, whereas CG’s item STM performance was within the control range but his order STM capacity was impaired. Finally, this distinction is also supported by recent neuroimaging studies showing that the retention of item information activates regions in superior and inferior temporal gyri, while the retention of order information activates a distinct network involving the right intraparietal sulcus (Majerus, Belayachi, et al., 2008; Majerus et al., 2010).

Furthermore, within item STM, some authors draw another distinction, between temporary storage of phonological and of lexical-semantic information, which they attribute to two separate limited-capacity buffers (Martin, Lesch, & Bartha, 1999; Martin, Shelton, & Yaffee, 1994). This distinction is based on neuropsychological case studies on brain-damaged patients with selective impairment of either phonological or lexical-semantic short-term storage (cases EA and AB from Martin et al., 1994; cases BN and TM from Verhaegen, Piertot, & Poncelet, 2013). The patients in these studies were either selectively impaired on phonological STM tasks (such as a rhyme probe task or a lexical decision task with phonologically related prime words), with preserved performance on lexical-semantic STM tasks (such as a category probe task or a lexical decision with semantically related primes), or vice versa.

These dissociations within the STM system have received very little attention in studies of age-related effects on STM in older adulthood. To our knowledge, only the study of Maylor, Voudsen, and Brown (1999) has explored this issue, looking at age-related differences in the production of order vs. item errors on a serial recall of letters. The authors showed that older participants produced more errors than younger participants, with no dissociation between item and order errors, suggesting that these two STM components may be equally affected by aging.

This study is an explorative study. First, we aimed at confirming the presence of an auditory-verbal STM decline in aging by assessing participants with a digit span task, a classical measure of STM, widely used in the literature (e.g., Belleville et al., 1996; Fisk & Warr, 1996; Grégoire & Van der Linden, 1997; Hester et al., 2004). Given that this typical STM task does not distinguish between item and order retention abilities, poor performance on this task in older adults could reflect deficits in item STM, order STM, or both. We argue that knowing whether all components of STM are affected equally by the aging process has interest for future evaluation of the auditory-verbal STM in older adults and for a better understanding of the eventual degradation. Therefore, participants were presented STM tasks specifically designed to measure order and item retention abilities separately. Within item STM, the phonological and lexical-semantic short-term storages were also assessed distinctly.

Moreover, the presence of STM decline in aging is often studied in participants older than 60 years of age (e.g., Belleville et al., 1996; Fisk & Warr, 1996; Peters et al., 2007). In our study, in order to explore the effects of aging over a wider range of stages in the lifespan, we presented our STM tasks to participants from 4 age groups: 45–54, 55–64, 65–74 and 75–84 years of age.

Serial order STM was assessed with a digit serial order recognition task and a serial order reconstruction task (animal race task). These tasks were chosen to maximize short-term retention of serial order and minimize the need to retain item information, since the items presented were of high lexical frequency, sampled from a closed pool of items, known in advance (i.e., the items used in a particular sequence were presented in advance, in ascending order), and provided to the participants at recall, so that the participants needed to focus only on retaining the order of presentation of the memoranda. By contrast, STM for item information was investigated using a word recognition task, and with a nonword delayed repetition task. These tasks were chosen to maximize the need for retention of item information and reduce the need to retain order information, since the items were sampled from an open pool of items, new on every trial, and of moderate lexical frequency or nonwords, whereas retaining the order of presentation of the items was not required. Phonological and lexical-semantic STM capacities were explored using a rhyme probe task and a category probe task, in which the participants respectively had to focus on whether the probe items rhymed with (phonological information) or belonged to the same semantic category (lexical-semantic information) as one of the items previously presented in a sequence.

All the tasks used in this study use auditory presentation, as is the case in many studies on STM decline in aging (e.g., Belleville et al., 1996; Fisk & Warr, 1996; Grégoire & Van der Linden, 1997; Hester et al., 2004; Peters et al., 2007). However, it is well known that older adults often present reduced hearing levels. One third of adults above the age of 70 have been shown to present some clinically significant hearing loss, and almost 100 percent have reduced hearing levels (e.g., Cruickshanks et al., 1998; see also the discussion of Surprenant, 2007). Hearing decline has been reported to proceed at a rate of 3 to 5 dB per decade before the age of 60 and of 9 to 15 dB thereafter (Cheesman, 1997; van Boxtel et al., 2000). Despite this clinical reality, Surprenant pointed out that only 25 percent of the studies exploring cognitive capacities in aging have considered hearing acuity as a factor in their experiments, or have even measured participants’ hearing status. Auditory-verbal STM capacities in older participants may have been underestimated as a result.

Many studies have indeed indicated a relationship between sensory status and cognitive functioning. For instance, Lindenberger and Baltes (Baltes & Lindenberger, 1997; Lindenberger & Baltes, 1994; Lindenberger & Ghisletta, 2009) have observed strong correlations, which increase with age, between vision and hearing status and performance on tasks designed to measure cognitive functioning. Moreover, Lunner (2003) highlighted the association between hearing impairment and STM performance by showing that improving older adults’ hearing using hearing aids could in turn improve their cognitive performance compared to same-age controls without hearing aids (see also discussions of Hoi Ning Ng, Rudner, Lunner, Syskind Pedersen, & Rönnberg, 2013; van Hooren et al., 2005).

The direct impact of impaired hearing on STM performance has been widely studied (e.g., Baldwin & Ash, 2011; Cervera, Soler, Dasi, & Ruiz, 2009; McCoy et al., 2005; Murphy, Craik, Li, & Schneider, 2000; Pichora-Fuller, Schneider, & Daneman, 1995; Piquado, Cousins, Wingfield, & Miller, 2010; Rabbit, 1968; Rabbit, 1991; Schneider & Pichora-Fuller, 2000; van Boxtel et al., 2000; Verhaegen, Collette, & Majerus, 2013; Wingfield, McCoy, Peelle, Tun, & Cox, 2006; Wingfield, Tun, & McCoy, 2005). McCoy et al. (2005) and Rabbit (1991) showed that older adults with mild auditory impairment recalled fewer words than older participants without hearing loss.

In order to distinguish the effects of aging from the effects of hearing status on STM, a number of studies compared recall performance of young and older participants, matched for hearing loss, either naturally or experimentally induced. Baldwin and Ash (2011), Murphy et al. (2000) and Pichora-Fuller et al. (1995) simulated age-related hearing loss in young participants by either decreasing the intensity of auditory stimuli (e.g., Baldwin & Ash, 2011) or by adding background noise during stimulus presentation (e.g., Murphy et al., 2000; Pichora-Fuller et al., 1995). When comparing the recall capacities of the younger participants to the capacities of the older ones, the authors showed that recall performance decreased both in young and older adults when stimulus presentation was degraded. Nevertheless, their manipulations did not remove the effects of aging. On the other hand, some other studies suggest that auditory impairment may explain age-effects in STM performance more than does aging (Cervera et al., 2009; Pichora-Fuller et al., 1995; van Boxtel et al., 2000; Verhaegen et al., 2013; Wingfield et al., 2006). Indeed, Verhaegen et al. (2013) and Wingfield et al. (2006) showed that when matched for hearing thresholds, young and older participants performed equally on auditory-verbal STM tasks (Verhaegen et al., 2013) or on an auditory comprehension task with syntactically simple short sentences (Wingfield et al., 2006), and that the overall performance of both auditory-reduced groups was worse than that of participants with good hearing. Moreover, Cervera et al. (2009) and van Boxtel et al. (2000) further found that after controlling for hearing thresholds with analyses of covariance or partial correlations, differences in STM performance between younger and older participants became non-significant. Finally, in another part of their study, Pichora-Fuller et al. (1995) showed that when the same memoranda were presented in a visual rather than an auditory form, younger and older participants performed at the same level. In conclusion, participants’ hearing status seems to be an important factor to take into consideration in STM assessment. In order to control whether participants’ hearing status had an impact on their performance on our auditory-verbal STM tasks, we also measured participants’ hearing thresholds with a pure-tone audiometer. We believe that considering this factor in an experiment is crucial given the importance of hearing difficulties in aging and has also an interest for future assessment of auditory-verbal STM wherein STM tasks are usually auditorily presented.

## Method

### Participants

Four groups of participants took part in the present study: (1) 20 participants between 45 and 54 years of age, (2) 22 participants between 55 and 64 years of age, (3) 22 participants between 65 and 74 years of age, and (4) 20 participants between 75 and 84 years of age. Participants responded to a questionnaire on their health and reported no history of neurological, cardiac, neuropsychological or psychiatric disorders, and no uncorrected or visual problems. Participants’ auditory acuity was measured via pure-tone audiometry, with 250, 500, 1000, 2000, 3000 and 4000 hertz (Hz) frequency tones presented to the right and left ears. We calculated bilateral mean pure-tone averages (PTAs) including each frequency as a measure of general hearing acuity (e.g., Cervera et al., 2009; van Boxtel et al., 2000). Mean PTAs were 10.75 dB HL (*SD* = 4.68) for the 45–54 age group, 17.80 dB HL (*SD* = 5.12) for the 55–64 age group, 23.94 dB HL (*SD* = 6.64) for the 65–74 age group and 31.42 dB HL (*SD* = 10.47) for the 75–84 age group. An analysis of variance (ANOVA) performed on the participants’ mean PTAs showed a significant effect of group, *F*(3, 82) = 31.46, *MSE* = 49.67, *p* < .001, η^2^_p_ = .90. Tukey’s HSD post hoc comparisons (*p* < .05) indicated that the 45–54 age group had lower hearing thresholds than the 55–64 age group (*p* = .009), who had lower hearing thresholds than the 65–74 age group (*p* = .02), who finally had lower hearing thresholds than the 75–84 age group (*p* = .005). Bilateral mean pure-tone audiometry thresholds for 250, 500, 1000, 2000, 3000 and 4000 Hz are presented in Table 1.

**Table 1:** Demographic data for the 45–54, 55–64, 65–75 and 75–84 year age groups.

Variable		45–54 years	55–64 years	65–74 years	75–84 years

Number of participants		20	22	22	20
Age (years)^a^		49.60 (3.70)	58.59 (3.08)	68.82 (3.05)	79.10 (3.28)
Gender (M/F)		6/14	9/13	8/14	7/13
Socio-economic background^b^	Low	.25	.27	.41	.40
	Middle	.45	.50	.36	.30
	High	.30	.23	.23	.30
Pure-tone thresholds (dB HL) ^a,c^
*250 Hz*		10.75 (6.29)	14.69 (6.23)	20.68 (7.57)	26.25 (7.76)
*500 Hz*		10.25 (6.06)	13.65 (6.25)	20.81 (7.25)	27.25 (8.66)
*1000 Hz*		11.25 (8.21)	12.69 (6.37)	19.20 (6.79)	28.13 (10.63)
*2000 Hz*		11.50 (6.90)	15.10 (7.50)	23.30 (7.54)	28.50 (11.28)
*3000 Hz*		11.75 (7.48)	20.00 (10.53)	29.32 (7.53)	36.63 (13.60)
*4000 Hz*		15.00 (8.11)	23.52 (9.75)	30.57 (8.79)	40.50 (13.34)
Mattis Scale (/144)^a^		143.45 (1.00)	142.45 (1.77)	140.27 (2.29)	140.65 (2.54)
Mill Hill (/33)^a^		25.45 (4.37)	25.59 (4.17)	24.41 (4.51)	26.20 (4.04)

*Note*. ^a^ Means and standard deviations in parentheses; ^b^ Proportion; ^c^ Bilateral mean pure tone audiometry thresholds.

None of the participants wore a hearing aid. Indeed, in this study, we aimed to control for the impact of reduced hearing on STM performance in natural conditions, without any hearing improvement via hearing aids. A number of studies (e.g., Lunner, 2003) have suggested that correction of hearing loss with hearing aids improves cognitive performance. Moreover, Wingfield et al. (2006) reported that two out of three elderly adults with hearing loss do not use hearing aids. We thus supposed that participants without hearing aids would be more representative of the elderly population.

Participants were also carefully screened for medication use. Participants taking antidepressants or other psychoactive medications were excluded from the study. Participants were given the Mattis Dementia Rating Scale (Schmidt, Freidl, Fasekas, Reinhart, & Grieshofer, 1994) and all of them performed above the cut-off score of 130/144. All participants were French native speakers. There were no reliable differences between the 4 groups in the distribution of socio-economic backgrounds. Participants were classified into 3 socio-economic levels: low, middle and high, according to the classification of Amos et al. (2003) which determines socio-economic level on the basis of participants’ years of schooling and profession. A chi-squared test showed no significant relationship between socio-economic background and group, χ^2^(6, *n* = 84) = 2.96, *ns*. All groups were also matched for vocabulary level: an ANOVA computed on the number of correct responses out of 33 on the Mill Hill test (Deltour, 1993) revealed that all age groups performed equally, *F*(3, 80) = 0.64, *MSE* = 18.33, *p* = *.59*.

Finally, cognitive processing speed was assessed via an odd/even judgment of digit task. In this task, 50 digits between 1 and 9 were presented in random order, centered on the computer screen, using the E-Prime 2.0 software (Psychology Software Tools). Participants had to indicate whether the digits were odd or even by pressing a designated “odd” or “even” key as quickly and accurately as possible. We performed an ANOVA on the log-transformed response latencies^1^. A significant group effect, *F*(3,80) = 35.25, *MSE* = .02, *p* < .001 = η^2^_p_.57, was observed, and Tukey’s HSD post hoc comparisons showed that the 45–54 age group responded faster than the 55–64 age group (*p* < . 001), which responded as quickly as the 65–74 age group (*p* = .35). The 75–84 age group was slower than the three younger groups (with the 45–65 age group: *p* < .001; with the 55–64 age group: p < .001; with the 65–75 age group: *p* = .005).

We chose to analyze the age-related effects on short-term memory capacities on four groups of 10 years each, between 45 and 85 years of age because we assumed that these age-related changes between different decades remain subtle. Moreover, as indicated above, the age-related decline of auditory acuity also proceeds per decade, at a rate of 3 to 5 dB per decade before the age of 60 and of 9 to 15 dB thereafter (Cheesman, 1997; van Boxtel et al., 2000). Therefore, we aimed at examining how these modifications of auditory acuity would interfere with short-term memory capacities.

The local research ethics committee approved the study, and all human data included in this manuscript was obtained in compliance with the Helsinki Declaration. All participants gave their informed consent. Demographic data are summarized in Table 1.

### Materials

#### General STM capacity: digit span task

A digit span task (Weschler, 1989) was used to assess general STM capacity. Participants were orally presented with sequences of digits, drawn from the numbers 1 to 9, and asked to recall the digits in the order of their presentation. The digits were read aloud by the experimenter and presented at a rate of one item per second. The sequences, which contained from 2 to 9 items, were presented in ascending order of length. There were 2 trials per sequence length. All 16 sequences were presented to each participant. One practice trial preceded the task and was not included in the scoring. We computed the percentage of sequences correctly recalled by pooling over all sequence lengths.

#### Order STM

##### Digit serial order recognition task

This task (from Majerus, Leclercq et al., 2009) consisted of the auditory presentation of lists of 3 to 8 digits (the targets), followed by the presentation of the same digit lists (the probes) but on two thirds of trials, the serial order of two adjacent items was exchanged. The participants were then asked to indicate their judgment as to whether or not the digits were presented in the same order in the two lists by pressing a designated key. Sequences of digits were presented in ascending order of length, with 6 trials per sequence length. The lists consisted of digits between 1 and 8. For list length 3 only the digits 1, 2, and 3 were used; for list length 4, only 1, 2, 3, and 4 were used; and so on for other list lengths. The end of the target list was signaled by a brief tone and was followed by the second list of digits. The position of the reversal was balanced across all possible pairs of serial positions. Before each list length, the participants were told that they would hear two lists of digits, and on each trial the digits present in the two sequences were presented to the participants in ascending order. The stimuli had been prerecorded by a female speaker in a sound-attenuating room and stored on a computer disk, and were presented via headphones connected to a PC, using E-Prime 2.0 software, at a comfortable hearing level (70 dB SPL) frequently chosen in auditory-verbal STM experiments (e.g., Majerus & D’Argembeau, 2011). We computed the percentage of correct “yes” and “no” responses for each sequence length. On this task, the need to retain item information was minimized since the digits are highly familiar and frequent characters, were known in advance, and remained the same both within each sequence length and between the target and probe sequences. By contrast, the task maximized the retention of order information, since the participants had to focus only on the digits’ order of presentation.

##### Serial order reconstruction: the animal race task

This task, adapted from Majerus, Poncelet, Greffe et al. (2006), consisted of the auditory presentation of sequences of animal names. At the end of each trial, the participants were given cards printed with colored pictures of the animal names presented during the sequence and asked to sort the cards according to their order of presentation, by ordering them on the desk from left to right. Stimulus recording and presentation procedures were the same as in the preceding task. Eight animal names (‘chat’, ‘loup’, ‘mouche’, ‘chien’, ‘ours’, ‘singe’, ‘coq’, ‘rat’, meaning cat, wolf, fly, dog, bear, monkey, rooster, rat) were used to form the lists, containing 4 to 8 items. The lists were again presented by increasing order of length, with 4 trials per sequence length. The participants were told at the beginning of each trial which items would be presented in the sequences. For each length, the same animal names were selected to construct the sequences: for instance, for sequences of length 4, the cat, the fly, the rooster and the wolf were used, and so on for the other sequence lengths. Only the cards for the animals actually presented on the trial were given to the participants. The 8 stimuli were monosyllabic and had high lexical frequency (> 16 000 occurrences per million; New, Brysbaert, Véronis, & Pallier, 2007) and a low age of acquisition (range: 13–24 months; Alario & Ferrand, 1999). We computed the percentage of items correctly recalled in their order of presentation by pooling over all sequence lengths. This task was designed to maximize the recruitment of serial order STM as the participants had to focus on the serial position in which each item occurred. On the other hand, the need for item STM was minimized, since the stimuli were known in advance, monosyllabic, had high lexical frequency, sampled from a limited pool of frequent names, and provided at the time of recall through the cards.

#### Item STM

##### Word recognition task

This task from Majerus, Metz-Lutz, Van der Kaa, Van der Linden, and Poncelet (2007), consisted of the auditory presentation of sequences of words. After each sequence, a new sequence comprised of all target stimuli and an equal number of distractor stimuli was presented. The participants had to decide whether each probe stimulus matched one of the stimuli in the target list and give their response by pressing a designated key. The stimuli were presented in sequences of between 2 and 5 items, presented in increasing order of length, with 4 trials per sequence length. Stimulus recording and presentation procedures were the same as for the preceding tasks. The end of the target list was indicated by a brief tone, which was followed by the probe list. Fifty-six targets and 56 negative probe words (distractors) were constructed. The items all had a CVC syllabic structure. The negative probe item differed from the target only by the initial consonant. Lexical frequency was similar for targets (*M* = 18 893 occurrences per million; Content, Mousty, & Radeau, 1990) and distractors (*M* = 17 545 occurrences per million; Content et al., 1990). We computed the percentage of correct responses on each task by pooling over trials and sequence lengths.

This task maximized the demands on memory for item information and minimized the need to retain serial order information, since the items were new on every trial, sampled from an open pool, and participants only had to focus on the items’ identity whereas their order of presentation did not matter.

##### Nonword delayed repetition task

This task was adapted from Majerus and Van der Linden (2003) and was comprised of 30 nonwords. Stimulus recording and presentation procedures were the same as for the preceding task. The stimuli had a CVC syllabic structure, and all were in accordance with respect to French phonotactic rules (Tubach & Boë, 1990). The stimuli were presented in random order. Each was presented in isolation and followed by a 7-second delay during which the participant counted aloud backwards in steps of 3, starting at 95. At the end of the delay, the experimenter tapped sharply on the desk, indicating that the participant should repeat the target nonword. The task began with 4 practice trials which were not included in the scoring. We determined the percentage of phonemes that were correctly recalled by pooling over trials.

To maximize retention requirements for item information, the stimuli were new on any trial. In order to reduce serial order memory requirements, only a single item had to be maintained in each trial. Moreover, all nonwords were monosyllabic and had the same CVC syllabic structure, further reducing processing requirements at the level of sequence information (i.e., the sequence structure was the same in each trial, only the identity of the phonemes varied between trials). Finally, the nonwords were recalled after a filled delay which hindered sequential rehearsal of the information that was to be recalled.

##### Phonological and lexical-semantic STM: rhyme and category probe tasks

Phonological STM was assessed with a rhyme probe task, while lexical-semantic STM was assessed with a category probe task. These tasks, drawn from Majerus, Van der Linden, Poncelet, and Metz-Lutz (2004), were based on the probe tasks of Martin et al. (1994; 1999). Sequences of 2 to 7 items were presented, followed by a probe word. In the rhyme condition, the participants were asked to judge whether the probe word rhymed with any item in the preceding list; in the semantic category condition, they were asked to judge whether the probe word belonged to the same semantic category as one of the words in the preceding sequence. Responses were given by pressing a designated key. Stimulus recording and presentation procedures were the same as for the preceding tasks. There were 6 trials each with sequence length 2 and 3, and 7 trials each for sequence lengths 4 to 7. Each serial position was probed equally often. For each sequence length and each condition, there were 2 non-matching probe trials and the remainder (4 for lengths 2 and 3, 5 for lengths 4 to 7) were matching probe trials. A greater number of matching probes was chosen because Majerus et al. (2004) showed in their pilot study that non-matching probes were very easily rejected, while the detection of matching items was more difficult and yielded more variable scores, thus increasing the sensitivity of the task. All words were bisyllabic and of medium lexical frequency (*M* = 1 692/million for the rhyme probe; *M* = 2 009/million for the category probe; Content et al., 1990). The categories probed were animals, body parts, clothes, flowers, fruit, furniture, kitchen utensils, professions, tools, vegetables, and transportation. The names of the categories were presented to the participants before the presentation of sequences of lengths 2 and 3, but not before longer sequences, in order to keep participants from using a visual strategy consisting in visually remembering the categories that had already been presented. Four additional trials were used as warm-ups and were not included in the scoring. We computed the percentage of correct yes/no answers on each task by pooling over trials and sequence lengths.

The rhyme probe task maximized demands on phonological STM. Indeed, a word’s phonological trace needs to remain activated in STM to allow the rhyme judgment. By contrast, the category probe task maximized the need to retain lexical-semantic STM information: the semantic traces need to be still activated in STM to allow the judgment of same/different semantic category membership (Majerus et al., 2004). Moreover, these two tasks both minimized the need to draw on serial order STM capacities, because the participants had to focus only on whether the probe word rhymed with or belonged to the same category as one of the words in the sequence, regardless of its sequential position. Moreover, Majerus, Poncelet, Greffe et al. (2006) showed that performance on these tasks did not correlate with performance on a serial order reconstruction task.

### Procedure

The whole study was conducted in French. Participants were tested individually in a quiet room. The order of the tasks was constant across participants: (1) Pure-tone audiometry, (2) Digit span task, (3) Word recognition task, (4) Rhyme probe task, (5) Animal race task, (6) Digit serial order recognition task, (7) Category probe task, (8) Nonword delayed repetition task, (9) Odd/even judgment task, (10) Mill Hill, (11) Mattis Dementia Rating Scale. The experiment took about 120 minutes to complete and was performed in a single session in participants from 45 to 64 years and in two sessions in participants from 65 to 84 years, in order to prevent interference with the results due to fatigue.

## Results

Table 2 gives the mean percentage of correct responses obtained for the 45–54, 55–64, 65–75 and 75–84 age groups on all STM tasks.

**Table 2:** Performance (mean percentage, standard deviation in parentheses) on the short-term memory tasks of the 45–54, 55–64, 65–75 and 75–84 age groups.

Task	45–54 years	55–64 years	65–74 years	75–84 years

Digit span	63.75 (13.54)	59.37 (8.76)	50.85 (10.79)	53.43 (10.43)
Digit serial order recognition	85.56 (7.94)	78.91 (11.23)	73.11 (15.05)	75.56 (6.66)
Serial order reconstruction (Animal race task)	68.61 (13.77)	68.28 (8.46)	69.94 (6.76)	62.39 (16.80)
Word recognition	92.05 (5.69)	88.23 (6.65)	86.61 (6.81)	82.50 (9.64)
Nonword delayed repetition	82.16 (14.23)	77.12 (10.09)	67.22 (13.12)	66.44 (13.18)
Rhyme probe	88.52 (5.83)	85.85 (7.19)	78.10 (14.57)	81.02 (7.60)
Category probe	81.93 (6.19)	81.30 (8.50)	71.59 (15.02)	74.89 (8.26)

We first performed analyses of variance (ANOVA) to check for age-related effects on the different STM tasks. When an age effect was found, because of the differing hearing status of the 4 age groups, we further aimed to control whether these age-related differences remained significant after performing analyses of covariance (ANCOVAS) on the results from the different STM tasks, using hearing thresholds (mean PTAs) as covariate, as in the study of Cervera et al. (2009).

### General STM capacity: digit span task

We performed an ANOVA on the percentage of correct responses on the digit span task. The analysis revealed an effect of age, *F*(3, 80) = 5.87, *MSE* = 120.20, *p* = .001; η^2^_p_ = .18. The age-related decline was confirmed by Tukey’s HSD post hoc comparisons (*p* < .05), but not in all comparisons. The post hoc tests indicated that the 45–54 age group performed better than the 65–74 (*p* = .002) and 75–84 age groups (*p* = .02), but that the performance of the 45–54 and 55–64 age groups was equivalent (*p* = .59), as was that of the 55–64, 65–74 and 75–84 age groups (55–64 and 65–74 age groups: *p* = .06; 55–64 and 75–84 age groups: *p* = .32; 65–74 and 75–84 age groups: *p* = .88). Furthermore, as indicated in Table 2, the effects of aging on this task are not linear. Indeed, the 75–84 age group had better results than the 65–74 age group but these differences are not significant.

**Figure 1:**
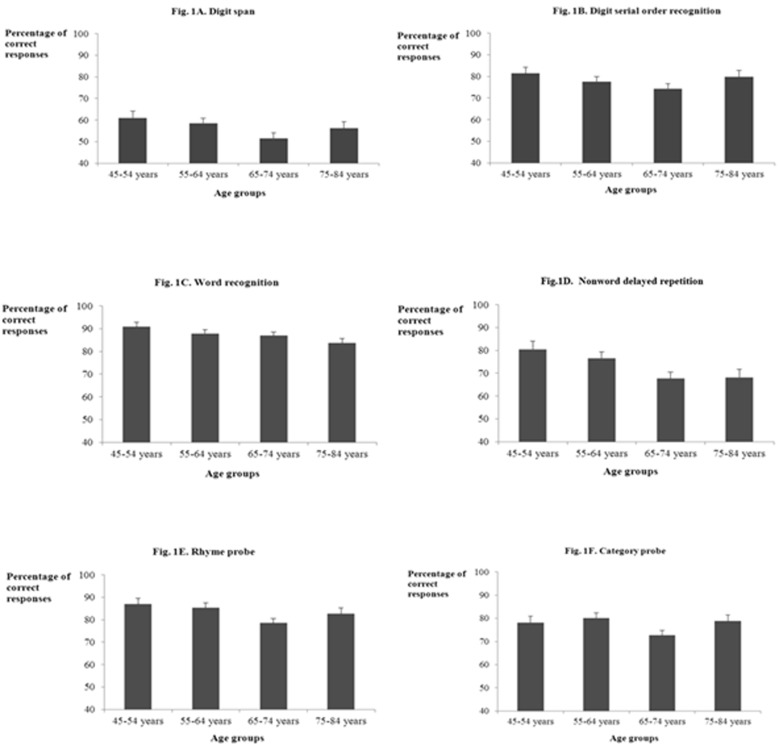
Adjusted means for the analyses of covariance performed on the short-term memory tasks with mean PTA as covariate, in the 45-54, 55-64, 65-75 and 75-84 age groups; Fig. 1A. Digit span; Fig. 1B. Digit serial order recognition; Fig. 1C. Word recognition; Fig. 1D. Nonword delayed repetition; Fig. 1E. Rhyme probe; Fig. 1F. Category probe.

The ANCOVA analysis on the percentage of correct responses on the digit span task, using mean PTAs as covariate, the results showed only marginally significant differences between the 4 age groups, *F*(3, 79) = 2.20, *MSE* = 118.31, *p* = *.09.* Thus, there were only marginally significant differences between the four age groups on the digit span task when hearing thresholds were controlled for. The adjusted means for the analyses of covariance performed on the STM tasks in the 45–54, 55–64, 65–75 and 75–84 age groups are presented in Figure 1.

### Order STM

#### Digit serial order recognition task

The ANOVA performed on the percentage of correct responses on the digit serial order recognition task^2^ showed an effect of age, *F*(3, 80) = 5.09, *MSE* = 118.10, *p* = .003; η^2^_p_ = .16. The age-related decrement was confirmed by Tukey’s HSD post hoc comparisons (*p* < .05), which indicated that that the 45–54 age group performed better than the 65–74 (*p* = .002) and 75–84 age groups (*p* = .02). The 45–54 and the 55–64 age groups performed equally well (*p* = .22), as did the 55–64, 65–74 and 75–84 age groups (55–64 and 65–74 age groups: *p* = .29; 55–64 and 75–84 age groups: *p* = .76; 65–74 and 75–84 age groups: *p* = .89). Moreover, as indicated in Table 2, the effects of aging on this task are not linear. Indeed, the 75–84 age group had better results than the 65–74 age group but these differences are not significant.

The ANCOVA performed on the percentage of correct responses on the digit serial order recognition task, using mean PTAs as a covariate, showed that differences between the 4 age groups were only marginally significant, *F*(3, 79) = 1.68, *MSE* = 111.22, *p* = *.06* (see Figure 1).

#### Serial order reconstruction: the animal race task

We performed an ANOVA on the mean percentage of correct responses on the serial order reconstruction task. The analysis showed no significant differences between the 4 age groups, *F*(3, 80) = 1.61, *MSE* = 143.00, *p* = *19.*

### Item STM

#### Word recognition task

An ANOVA performed on the mean percentage of correct responses on the word recognition task^2^ assessed age effects. The results showed significant differences between the 4 age groups, *F*(3, 80) = 5.87, *MSE* = 53.30, *p* = .001; η^2^_p_ = .18. Tukey’s HSD post hoc comparisons (*p* < .05) confirmed the age-related decline between the 45–54 and 75–84 age groups, with the 45–54 age group performing better than this older group (*p* < .001). By contrast, the 45–54, 55–64 and 65–74 age groups performed equally (45–54 and 55–64 age groups: *p* = .36; 45–54 and 65–74 age groups: *p* = .09; 55–64 and 65–74 age groups: *p* = .88), and the 55–64 and 65–74 age groups did not significantly differ from the 75–84 age group (55–64 and 75–84 age groups: *p* = .07; 65–74 and 75–84 age groups: *p* = .29). Moreover, as indicated in Table 2, the effects of aging on this task are not linear. Indeed, the 75–84 age group had better results than the 65–74 age group but these differences are not significant.

The ANCOVA performed on the percentage of correct responses on the word recognition task using hearing thresholds as covariate showed no significant differences between the 4 age groups, *F*(3,79) = 1.56, *MSE* = 53.56, *p* = .20. Thus, when hearing capacities were controlled for, there was no age-related effects on the word recognition task (see Figure 1).

#### Nonword delayed repetition

The ANOVA performed on the square root-transformed percentage of phonemes correctly repeated^3^ revealed a significant group effect, *F*(3, 80) = 9.54, *MSE* = 2.78 * 10^6^, *p* < .001, η^2^_p_ = .26 (see Table 2). Tukey’s HSD post hoc comparisons (*p* < .05) indicated an age-related decline, between 45–54 age group and the two older age groups (65–74 and 75–84 years) with the 45–54 age group performing better than these two groups (45–54 and 65–74 age groups: *p* < .001; 45–54 and 75–84 age groups: *p* < .001). By contrast, the 45–54 and the 55–64 age groups performed equally (*p* = .33). Moreover, the 55–64 age group performed better than the 65–74 (*p* = .04) the 75–84 age group (*p* = .03). The 65–74 and the 75–84 age groups performed equally (*p* = 1.00).

The ANCOVA on the square root-transformed percentage of phonemes correctly repeated on the nonword delayed repetition task with mean PTAs as covariate indicated that the age-related differences remained significant, even when mean PTA was controlled for, *F*(3,79) = 3.55, *MSE* = 2789492, *p* = .02, η^2^_p_ = .12. As in the ANOVA, Tukey’s HSD post hoc comparisons (*p* < .05) indicated that the 45–54 age group performed better than the two oldest groups, of 65–74 and 75–84 years (45–54 and 65–74 age groups: p < .001; 45–54 and 75–84 age groups: *p* < .001). By contrast, the 45–54 and the 55–64 age groups performed equally (*p* = .32). Moreover, the 55–64 age group performed better than the 65–74 (*p* = .04) the 75–84 age group (*p* = .03). The 65–74 and the 75–84 age groups performed equally (*p* = 1.00) (see Figure 1).

#### Phonological and lexical-semantic STM

##### Phonological STM: rhyme probe task

An ANOVA on the percentage of correct responses on the rhyme probe task was performed 2. The results indicated an effect of age, *F*(3, 80) = 5.08, *MSE* = 91.10, *p* = .003, η^2^_p_ = .16. Tukey’s HSD post hoc comparisons (*p* < .05) showed that the 45–54 and 55–64 age groups performed better than the 65–74 age group (45–54 and 65–74 age groups: *p* = .004; 55–64 and 65–74 age groups: *p* = .04). The 65–74 and the 75–84 age groups performed equally (*p* = .77) and the 75–84 age group did not significantly differ from the 45–54 (*p* = .81) and 55–64 age groups (*p* = .39) either. Moreover, as indicated in Table 2, the effects of aging on the rhyme probe task are not linear. Indeed, the 75–84 age group had better results than the 65–74 age group but these differences are not significant.

The ANCOVA performed on the percentage of correct responses on the rhyme probe task using mean PTAs as covariate indicated that after controlling for hearing capacity, the age effect was marginally significant, *F*(3,79) = 2.40, *MSE* = 91.01, *p* = *.07* (see Figure 1).

##### Lexical-semantic STM: category probe task

The ANOVA conducted on the percentage of correct responses on the category probe task^2^ revealed a significant difference between the 4 age groups, *F*(3,80) = 5.20, *MSE* = 103.50, *p* = .002, η^2^_p_.16. As on the rhyme probe task, Tukey’s HSD post hoc comparisons (*p* < .05) showed that the 45–54, 55–64 and the 75–84 age groups performed equally (45–54 and 74–84 age groups: *p* = .13; 55–64 and 74–84 age groups: *p* = .20) and that both the 45–54 and the 55–64 age groups performed better than the 65–74 age group (45–54 and 65–74 age groups: *p* = .01; 55–64 and 65–74 age groups: *p* = .01). Finally, the 65–74 and the 75–84 age groups did not significantly differ from each other (*p* = .73). Moreover, as indicated in Table 2, the effects of aging on this task are not linear. Indeed, the 75–84 age group had better results than the 65–74 age group but these differences are not significant.

The ANCOVA performed on the percentage of correct responses on the category probe task using mean PTAs as covariate indicated that, when hearing capacity was controlled for, the age effect was marginally significant, *F*(3, 79) = 2.33, *MSE* = 97.85, *p* = *.08* (see Figure 1).

### Correlational analyses

Thus, audition capacities seem to have an influence on participants’ short-term memory performance. In order to analyze the relation between the covariate (participants’ bilateral mean pure tone audiometry thresholds) and the participants’ scores on the short-term memory tasks, we performed correlational analyses between participants’ mean PTAs and their results on the short-term memory tasks. The results indicated on Table 3 show that mean PTAs were correlated with the scores on the short-term memory tasks. However, age is also related with short-term memory abilities. We reinforce that statement by analyzing the correlations between participants’ age and their scores in short-term memory tasks. The results indicated on Table 3 show that age is significantly correlated with participants’ short-term memory performance.

**Table 3:** Correlations between participants’ bilateral mean pure tone audiometry thresholds (mean PTAs) and their scores on the short-term memory tasks as well as between participants’ age and their scores on the short-term memory tasks.

Variables	Correlations with participants’ mean PTAs	Correlations with participants’ age

Digit span	*r* = −.37, *p* = .001	*r* = −.35, *p* = .001
Digit serial order recognition	*r* = −. 41, *p* < .001	*r* = −.36, *p* = .001
Word recognition	*r* = −.38, *p* < .001	*r* = −.35, *p* = .001
Nonword delayed repetition	*r* = −.42, *p* < .001	*r* = −.50, *p* < .001
Rhyme probe	*r* = −.31, *p* = .004	*r* = −.35, *p* = .001
Category probe	*r* = −.39, *p* < .001	*r* = −.36, *p* = .001

## Discussion

Age-related effects on STM have been generally studied using classical serial recall tasks (e.g., Belleville et al., 1996; Bopp & Verhaeghen, 2003; Fisk & Warr, 1996; Grégoire & Van der Linden, 1997; Hester et al., 2004; Kaulser, 1994; Peters et al., 2007), whereas more recent conceptions of verbal STM present it as a multicomponent system, in which each process can be selectively assessed and impaired. In these conceptions, a separation is postulated between the short-term storage of item information and the short-term storage of serial order information (Burgess & Hitch, 1999; Gupta, 2003; Henson, 1998; Majerus, Heiligenstein et al., 2009; Majerus, Leclercq et al., 2009; Majerus, Poncelet, Elsen et al., 2006; Majerus, Poncelet, Greffe et al., 2006; Majerus et al., 2008). Moreover, within item STM, another distinction is made between the short-term storage of phonological and lexical-semantic information (Martin et al., 1994; 1999). To our knowledge, the exploration of the effects of aging on these different STM components has received very little attention (for an exception, Maylor et al., 1999).

Therefore, the STM decline observed in older adults may be due to difficulties in item STM, order STM or both. Thus, in this study, in addition to a classical span task assessing both item and order STM, we assessed STM capacities for retaining order information using a serial order recognition task and a serial order reconstruction task (the animal race task), and item STM using a word recognition task and a nonword delayed repetition task. The subcomponents of phonological and lexical-semantic STM were evaluated with rhyme and category probe tasks respectively.

We sought to analyse the effects of aging on auditory-verbal STM not only in participants above 60 years of age but over a range of stages in the adult lifespan. We thus presented our tasks to participants in 4 age groups: 45–54, 55–64, 65–74 and 75–84 years of age.

This study confirmed the presence of an age-related decline on the classical forward digit span task. Moreover, the results showed that item and order STM, as well as phonological and lexical-semantic STM were affected by the aging process. Indeed, the results showed significant differences between the age groups on the digit serial order recognition task, the word recognition task, the nonword delayed repetition task and on the rhyme and the category probe tasks. The differences were especially observed between the youngest 45–54 age group and the two oldest groups (65–74 and 75–84 years of age), or between the 45–54 age group and only one of these two older groups. Moreover, the age effects on these STM components are not always linear. On the digit span task, the digit serial order recognition task and on the rhyme and category probe tasks, as indicated on the Table 2, the oldest group of 75 to 84 year old participants perform better than the 65 to 74 year old participants but the differences were not statistically significant.

However, given that older adults often present hearing loss (e.g., Cheesman, 1997; Cruickshanks et al., 1998; Surprenant, 2007; van Boxtel et al., 2000), we also aimed to control whether age-related hearing decline may account for the age-related differences observed in our STM tasks. We thus performed analyses of covariance on the different component of STM tasks, using mean PTAs as covariates, as Cervera et al. (2009) did on their classical recall tasks. After hearing status was controlled for with an ANCOVA, the significant differences between age groups disappeared on all STM tasks presented in our study, with the exception of the nonword delayed repetition task. This exception is discussed below. However, even if they were not statistically significant, the results of the digit span task, the digit serial order recognition task and on the rhyme and category probe tasks were marginally significant. These results indicate that there is an effect of aging on STM capacities. However, the fact that the differences between the four age-groups become statistically non-significant when mean PTAs are controlled for with an analysis of covariance leads us to conclude that age-related hearing loss is an important explanatory factor of STM difficulties in aging.

These findings add to a growing body of literature highlighting the impact of hearing loss on auditory-verbal STM capacities in older adults (e.g., Baldwin & Ash, 2011; Cervera al., 2009; McCoy et al., 2005; Murphy et al., 2000; Pichora-Fuller et al., 1995; Piquado et al., 2010; Rabbit, 1968; Rabbit, 1991; Schneider & Pichora-Fuller, 2000; van Boxtel et al., 2000; Verhaegen et al., 2013; Wingfield et al., 2005; Wingfield et al., 2006). Moreover, with exception of the nonword delayed repetition task, these results seem to be consistent with the results of Cervera et al. (2009), Pichora-Fuller et al. (1995), van Boxtel et al. (2000) and Verhaegen et al. (2013), which suggest that the decline in auditory-verbal STM is explained in most part by the participants’ hearing status degradation. The results we present here suggest that this assumption may apply to all components of auditory-verbal STM between the ages of 45 and 84.

As indicated above, the only task where the age-related differences remained significant even after having controlled the participants’ hearing statuses was the nonword delayed repetition task. We assume that these results are explained by the fact that this nonword repetition task requires different processes than the other auditory-verbal STM tasks, which are differently affected by aging. Indeed, the nonword repetition task involves an additional counting task. It cannot be totally excluded that the participants tried to maintain the memoranda while counting aloud by threes (although they were instructed not to), and therefore resorted to a combination of high demands on both storage and processing as well as attentional and executive functions. Executive functions have been shown to be affected by aging in many studies (e.g., Belleville, Rouleau, & Caza, 1998; Brink & McDowd, 1999; Gaeta, Friedman, Ritter, & Cheng, 2001; Kane, May, Hasher, Rahhal, & Stoltzfus, 1997; Milham et al., 2002). Tun, McCoy, and Wingfield (2009) found similar results in an experiment which also involved a counting sub-task in the course of a serial recall task, presented to older and young hearing-matched participants as well as to young and older participants with good hearing. The authors observed both hearing and age effects. By contrast, the other tasks presented in our study were passive auditory-verbal STM tasks, which required participants only to decode and maintain auditory memoranda, i.e., simple focusing on target stimuli. Oberauer (2001) showed that these latter processes are preserved in the elderly population. This may explain why we did not find additional effects of aging on these tasks.

The results in this study indicate that both age and audition, and probably many other age-related factors not explored here, are explanatory factors of short-term memory difficulties. These hypotheses are reinforced by the significant correlations between participants’ auditory thresholds and their scores on the short-term memory tasks and between participants’ age and their short-term memory performance.

We would also like to note here that the present results are only valid for the ‘passive’ immediate reproduction tasks used in the present study. It is likely that age effects on more complex STM memory tasks with higher executive load (known as ‘working memory tasks’) will persist. Hearing status effects may nevertheless also affect these working memory tasks, in addition to aging. In line with this assumption, Tun et al. (2009) observed both age and hearing status effects on a dual task combining serial recall and visual target pursuit tasks.

In order to tease apart the effects of hearing loss from other aging effects on STM, a more powerful design would compare young and older adults with good hearing to young and older adults with reduced hearing levels, matched for hearing thresholds as in Murphy et al. (2000), Tun et al. (2009), and Wingfield et al. (2006), but on tasks assessing all STM components, as here. Moreover, it would have also been of interest to compare the results in our auditory verbal STM tasks with performance on the same tasks using visual presentation—using written stimuli—in order to explore whether or not these outcomes are specific to the auditory modality (see Pichora-Fuller et al., 1995). However, Baltes and Lindenberger (1997) and Lindenberger and Baltes (1994) also reported strong correlations between vision and cognitive functioning in aging. Therefore, the results of such a study could be similar to those found here with auditory presentation.

STM decline in older adults has been related to several cognitive factors such as slowed processing speed (e.g., Salthouse, 1996), a lack of inhibitory control (e.g., Persad, Abeles, Zacks, & Denburd, 2002), increased sensitivity to interference (e.g., Oberauer & Kliegl, 2001), and context-item binding deficits (e.g., Oberauer, 2005). The results presented here indicate that hearing loss was also an important explanatory factor of poor STM performance in aging.

In conclusion, we found age-related effects on all components of auditory-verbal STM. However, when the participants’ hearing thresholds were controlled for with an analysis of covariance, the differences between age groups became non-significant. Thus, our results indicate that hearing impairment may be an important contributing factor in the impairment of auditory-verbal STM processes between the ages of 45 and 84. Given that age-related hearing loss is a pervasive and global health concern, this study highlights the need to take this factor into account when analyzing age-related decline in STM. Indeed, verbal STM capacities, which generally assessed with auditory-verbal span tasks, may be underestimated in this population due to their age-related decline in hearing abilities.

## Competing Interests

The authors declare that they have no competing interests.

## Footnotes

On the odd/even judgment of digit task, the data were log-transformed to meet the assumption of normality.

On the digit serial order recognition task, the word recognition task, and the rhyme and category probe tasks, the assumption of normality was not met for score distributions. No transformation made it possible to meet the assumption of normality. Therefore, analyses were also run using nonparametric Kruskal-Wallis tests. The results were identical.

For the results of the nonword delayed repetition task, a square root transformation was applied to meet the assumption of normality.
